# Uncovering somatic mosaic variants of *PIK3CA*-related overgrowth disorders – three cases with different clinical presentations

**DOI:** 10.3389/fgene.2024.1484651

**Published:** 2025-01-13

**Authors:** M. Tooming, P. Mertsina, T. Kahre, R. Teek, I. Vainumäe, S. Lilles, M. H. Wojcik, P. Ilves, K. Õunap

**Affiliations:** ^1^ Genetics and Personalized Medicine Clinic, Institute of Clinical Medicine, University of Tartu, Tartu, Estonia; ^2^ Genetics and Personalized Medicine Clinic, Tartu University Hospital, Tartu, Estonia; ^3^ Children’s Clinic, Institute of Clinical Medicine, University of Tartu, Tartu, Estonia; ^4^ Children’s Clinic, Tartu University Hospital, Tartu, Estonia; ^5^ Divisions of Newborn Medicine and Genetics and Genomics, Department of Pediatrics, Boston Children’s Hospital, Harvard Medical School, Boston, MA, United States; ^6^ Manton Center for Orphan Disease Research, Division of Genetics and Genomics, Department of Pediatrics, Boston Children’s Hospital, Harvard Medical School, Boston, MA, United States; ^7^ Broad Center for Mendelian Genomics, Broad Institute of Massachusetts Institute of Technology and Harvard, Cambridge, MA, United States; ^8^ Department of Radiology, Institute of Clinical Medicine, University of Tartu, Tartu, Estonia; ^9^ Radiology Clinic, Tartu University Hospital, Tartu, Estonia

**Keywords:** *PIK3CA*-related overgrowth syndrome, *PIK3CA* somatic mosaic variants, overgrowth syndrome, next-generation sequencing-based genomic profiling, *PIK3CA*, *PIK3CA* somatic mutation

## Abstract

**Introduction:**

*PIK3CA* related disorders (PRD, OMIM: *171834) are genetic disorders resulting from pathogenic somatic mosaic variants in the *PIK3CA* gene, which encodes a protein crucial for regulating cell growth and division. PRD typically manifest during the post-zygotic phase, leading to a broad spectrum of overgrowth and vascular malformations affecting various body regions.

**Methods:**

Conventional diagnostic methods struggle to detect and confirm pathogenic PIK3CA gene variants due to the mosaic nature of these disorders and the limited accessibility of affected tissues. In this study, we conducted comprehensive genomic profiling on a cohort of individuals with PRD to address these diagnostic challenges.

**Results:**

Our analysis revealed significant diagnostic challenges posed by somatic mosaicism in PRD. The comprehensive genomic profiling allowed for the meticulous evaluation of potentially pathogenic gene variants in affected individuals and their corresponding tissues.

**Discussion:**

Our findings advocate for the adoption of comprehensive genomic profiling in clinical practice to improve the detection and management of PRD. This approach can enhance patient care by providing a more accurate diagnosis and better understanding of the genetic underpinnings of PRD.

## 1 Introduction

*PIK3CA*-related disorders (PRD, OMIM: *171834) are a group of genetic conditions that lead to the overgrowth of various body parts due to post-zygotic somatic changes in the gene encoding phosphatidylinositol-3-kinase (PI3K) catalytic subunit alpha (p110alpha). Estimating the prevalence of PRD poses challenges due to variations in ascertainment methods and the wide range of phenotypic manifestations observed (Mirzaa et al., 1993).

The severity of PRD varies considerably, even among individuals harbouring identical genetic alterations. While some patients exhibit mild symptoms that can be effectively managed, others experience more severe and debilitating symptoms, necessitating ongoing medical care. Currently, no cure is available for PRD, and treatment mainly revolves around symptom management and preventing complications (Canaud et al., 2021).

Genetic testing plays a pivotal role as a diagnostic tool for PRD by facilitating the identification of pathogenic *PIK3CA* gene variants, which inform treatment decisions. However, clinical diagnosis is hindered due to disease variability, overlapping phenotypes such as *Proteus* Syndrome or PTEN Hamartoma Tumour Syndrome, and tissue mosaicism, which can pose challenges in confirming pathogenic gene variants (Keppler-Noreuil et al., 2015).

We therefore describe three cases of PRD identified at our clinical center, highlighting the diverse clinical manifestations and unique approaches needed to diagnose this condition.

## 2 Materials and methods

Informed consent was obtained from the parents or guardians of all patients with, additional consent from two families to publish their photographs. The study was approved by the Research Ethics Committee of the University of Tartu (278/T-19, 288/M-17, 340/M-17, 372/M-8 and 387/M-15).

DNA was extracted from the patients’ whole blood, and fibroblast cell cultures were derived from affected skin biopsies, skin biopsy or affected lymphangioma tissue. Sanger sequencing was employed to analyse the presence of the *AKT1* pathogenic gene variant p.Gly17Lys.

Next-Generation Sequencing (NGS) libraries were performed using TruSight One (TSO; 4,813 genes), TruSight One Expanded (TSOE; 6,699 genes) or TruSight Oncology 500 (TSO500; 523 genes) sequencing panels by the manufacturer’s protocols (Illumina Inc.). NGS was carried out on NextSeq 500 platform with NextSeq 500/550 High Output Kit v2.5 (Illumina Inc.). Reads were aligned to reference genome hg19 by Burrows-Wheeler Aligner (BWA) (Li and Durbin, 2009), and variants were called by Genome Analysis Toolkit (GATK) (McKenna et al., 2010) tools using BWA Enrichment v2.1 workflow or Dragen v4 server using TruSight Oncology 500 v.2.1.1 software Illumina (Illumina Inc.). Variants from VCF files were annotated by an in-house variant annotation pipeline involving Annovar (Wang et al., 2010), SnpSift (Ruden et al., 2012) and GATK (McKenna et al., 2010). CNV detection was carried out using CoNIFER software (Krumm et al., 2012).

Additionally, trio exome sequencing (ES) was performed on DNA extracted from whole blood, enriched using the SureSelect XT Human All Exon V5 kit (Agilent Technologies), and sequenced on an Illumina HiSeq 4000 platform (Illumina Inc.). Library preparation and a sequencing run were performed by GenomeScan B.V. Parents–offspring trio WES was performed. Raw sequencing reads from FASTQ files were aligned to the hg19 reference genome using BWA (Li and Durbin, 2009). Bioinformatic processing, variant calling, and annotation was done following GATK best practice guidelines using Picard (Picard Tools - By Broad Institute, 2023), GATK (McKenna et al., 2010), Annovar (Wang et al., 2010) and SnpSift (Ruden et al., 2012) software. CNVs were called using CoNIFER software (Krumm et al., 2012).

## 3 Results

**FIGURE 1:**
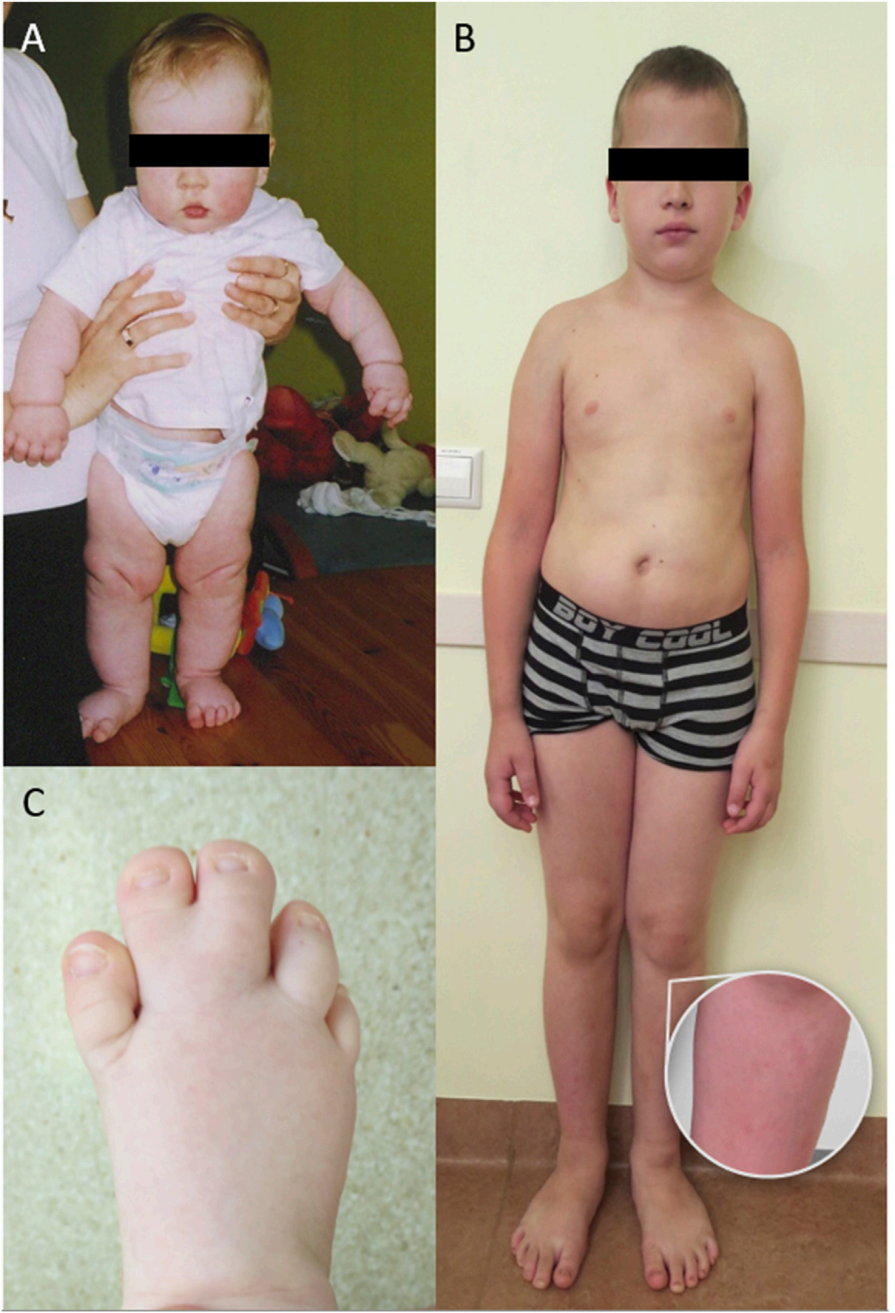
**(A)** Patient 1 at the age of 8 months, note high forehead, large head and *cutis laxa*; **(B)** Patient 1 at the age of 11 years, note high forehead, large head, body asymmetry, large feet and cutaneous port-wine stains on legs; **(C)** Patient 2 at the age of 1 year with macrodactyly of T2-3.

**FIGURE 2:**
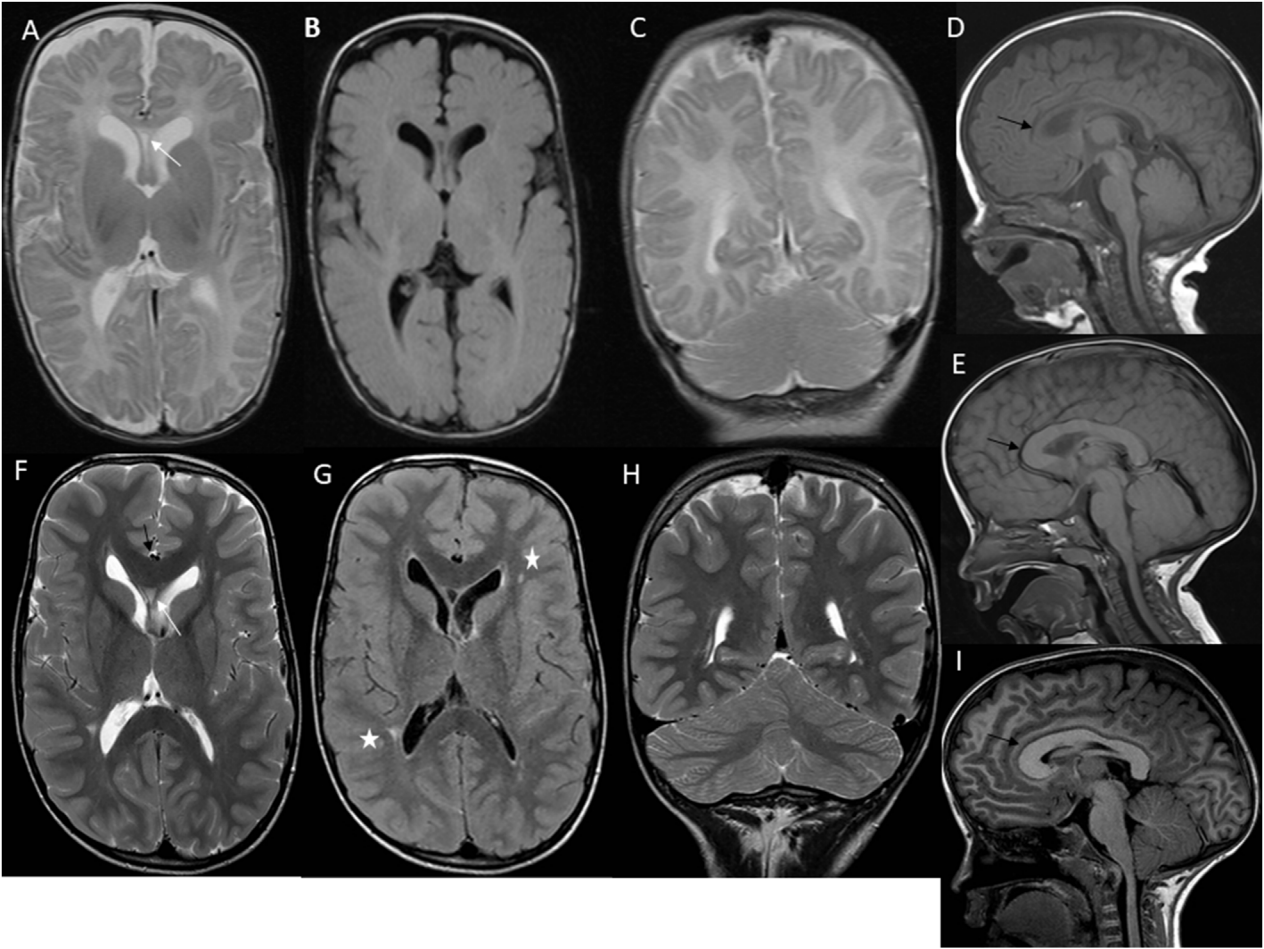
First case imaging series at different ages. At 2 months, imaging reveals megalencephaly, mild ventriculomegaly, and asymmetric development of cerebral and cerebellar hemispheres, along with a septum pellucidum cyst (white arrow) **(A–C)**. Sagittal T1 W image **(D)** shows an extended frontooccipital distance (150 mm, >97th percentiles) and an elongated corpus callosum (black arrow) (anteroposterior diameter 63 mm, >97th percentiles) with normal thickness. At 3 years, there is a persistent elongation of the frontooccipital distance and a thick corpus callosum (>97th percentiles) (black arrow) **(E)**. At 7 years, axial T2 W TSE image **(F)** shows ongoing megalencephaly, pronounced ventriculomegaly, asymmetric cerebral hemispheres, a small septum pellucidum cyst (white arrow), and a thick corpus callosum (black arrow) with normal myelination. Axial T2 W FLAIR image **(G)** highlights asymmetric ventriculomegaly and high signal focal changes in the periventricular white matter (white asterisks). Coronal T2 W TSE image **(H)** and sagittal T1 W image **(I)** reveal persistent asymmetry in cerebral and cerebellar hemispheres, extended frontooccipital distance (202 mm, >97th percentiles), mild cerebral tonsillar herniation (black arrow), and significant elongation and thickness of the corpus callosum (anteroposterior diameter 93 mm, >97th percentiles).

Since 2015, we have diagnosed three patients with PRD at our center. The first case involves a 19-year-old male (Table 1. Patient 1) presenting with macrocephaly, port-wine stains, low-set ears, high palate, large hands and feet, and mild to moderate intellectual disability (Figures 1A, B). Brain imaging studies revealed significant abnormalities: axial T2 turbo spin echo (TSE) weighted (W) (Figure 2A) and axial T2 W Fluid attenuated inversion recovery (FLAIR) (Figure 2B) images at 2 months showed megalencephaly with mild ventriculomegaly, asymmetric cerebral hemispheres, and a small septum pellucidum cyst. Coronal T2 W TSE image (Figure 2C) highlighted asymmetry in cerebral and cerebellar hemispheres. Sagittal T1 W image (Figure 2D) revealed a prolonged frontooccipital distance and an elongated corpus callosum while maintaining average thickness (Garel et al., 2011).

**TABLE 1:** Summary of clinical findings and *PIK3CA* variants.

Patient ID		Patient 1	Patient 2	Patient 3
Sex		M	F	F
Clinical symptoms, physical findings	Cutaneous	Port-wine stains		Lymphangiomas on the left thigh and both upper limbs
	Neurological	Mild to moderate intellectual disability	Normal development	Normal development
	Craniofacial	Macrocephaly, low-set ears, high palate		
	Internal organs			Multicystic lymphangiomas in abdominal cavity
	Other	Large hands and feet	Macrodactyly of the right foot T2-3	Antenatal cystic formation in the pelvis, syndactyly of T3-4 on the right and left foot, Macrodactyly of left foot
*PIK3CA* variant		NM_006218.4:c.2740G > A, p.Gly914Arg	NM_006218.4:c.1636C > A, p.Gln546Lys	NM_006218.4:c.1624G > A, p.Glu542Lys
VAF (%); sequencing depth (x) and (biopsy location)		23%; 205x (fibroblast culture derived from a skin biopsy obtained from the right leg)	10%; 51x (fibroblast culture derived from a skin biopsy between right foot T2-3)	0.8%; 1,236x (skin biopsy obtained from the left hip region)
				7.1%; 1,377x (biopsy of lymphangioma obtained from the left hip region)
				9%; 1,144x (biopsy of lymphangioma obtained from the left hip region)
*PIK3CA* domain		Kinase	Helical	Helical
Pathogenicity		P	LP	P
Hotspot variant		N	N	Y

VAF, variant allele frequency; P, pathogenic; LP, likely pathogenic; Y, yes; N, no.

Follow-up imaging at 3 years (Figure 2E) showed persistent elongation of the fronto-occipital distance and increased corpus callosum thickness. At 7 years, axial T2 W TSE images (Figure 2F) confirmed ongoing megalencephaly, mild ventriculomegaly, asymmetric cerebral hemispheres, a small septum pellucidum cyst, and a thick corpus callosum with normal myelination. Axial T2 W FLAIR image (Figure 2G) showed asymmetric ventriculomegaly and high signal focal changes in the periventricular white matter. Coronal T2 W TSE image (Figure 2 H) and sagittal T1 W image (Figure 2 I) at 7 years revealed asymmetry in cerebral and cerebellar hemispheres, extended frontooccipital distance, mild cerebral tonsillar herniation, and significant elongation and thickness of the corpus callosum. Trio ES of the patient’s blood failed to identify any pathogenic variants. Subsequently, a fibroblast culture derived from a skin biopsy of the affected tissue from the right leg was analyzed using the TSO gene panel, leading to the discovery of a heterozygous *de novo* mosaic pathogenic variant in the *PIK3CA* gene, specifically p.Gly914Arg, with a variant allele frequency (VAF) of 23% at a sequencing depth of 205x.

**FIGURE 3:**
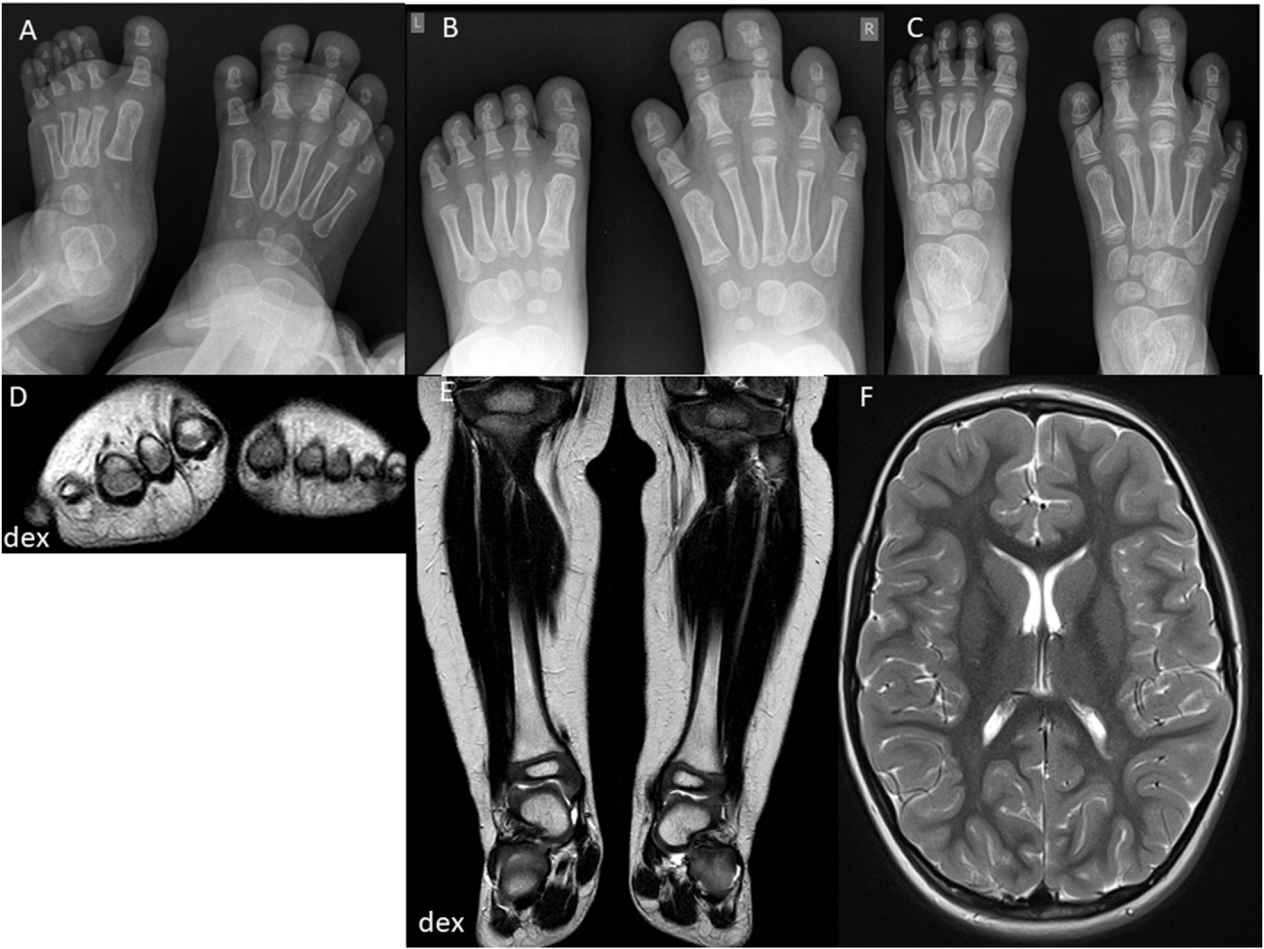
Second case imaging series at different ages. At 1.5 years, X-ray images **(A)** show increased size and length of metatarsal bones II-IV and toe phalanges II-IV on the right side, with syndactyly of the second and third toes. MRI **(D)** reveals enlarged metatarsal bones II-IV and increased thickness of plantar and dorsal soft tissues on the right foot. Coronal MRI **(E)** shows symmetric legs and heels with equal tibial lengths and consistent soft tissue thickness. At 2.5 years, X-ray images **(B)** indicate proportional growth in both feet, with consistent enlargement and elongation of metatarsal bones II-IV and toe phalanges II-IV on the right side, advanced bone age, and persistent syndactyly of the second and third toes. At 4 years, X-ray images **(C)** reveal further growth and advanced bone age in metatarsal bones and toes II-IV on the right side, with persistent syndactyly. MRI **(F)** shows normal and symmetrical cerebral structures and symmetrical development of other body parts without signs of malignancy.

The second case involves a 9-year-old female (Table 1. Patient 2) presenting with right foot macrodactyly of T2-3 (Figure 1C). Foot X-ray images at 1.5 years reveal enlarged and elongated II-IV metatarsal bones and phalanges, along with syndactyly of the second and third toes on the right side. The left foot exhibits normal morphology (Figure 3A). At 2.5 years, both feet showed proportional growth, but the right side had proportional enlargement and elongation of II-IV metatarsal and phalange bones, with advanced bone age in toes II-IV. Persistent syndactyly was observed in the second and third toes on the right (Figure 3B). X-rays at 4 years showed ongoing growth and advanced bone age in metatarsal bones and toes II-IV on the right, with persistent syndactyly (Figure 3C). MRI at 1.5 years revealed larger metatarsal bones II-IV on the right and increased thickness of plantar and dorsal soft tissues (Figure 3D). Coronal MRI at 1.5 years showed symmetric legs and heels with equal tibial length and consistent soft tissue thickness (Figure 3E). Brain MRI at 4 years showed normal cerebral structures and symmetrical development of other body parts without malignancy (Figure 3F). Due to a clinical suspicion of *Proteus* syndrome, only somatic testing was performed. Sanger sequencing was used to analyze a fibroblast culture derived from a skin biopsy taken from between the second and third toes of the right foot; no blood sample was tested. This analysis did not identify the pathogenic p.Glu17Lys variant in the *AKT1* gene. TSOE gene panel testing was performed on the same sample. This analysis revealed a heterozygous mosaic pathogenic variant in the *PIK3CA* gene, specifically p.Gln546Lys, with a VAF of 10% at a sequencing depth of 51x.

The third case is a 4.5-year-old female (Table 1. Patient 3) presenting with lymphatic and vascular malformations (lymphangiomas) on the left thigh and both upper limbs, cystic formation found antenatally, retroperitoneally and in the small pelvis, syndactyly of T3-4 on both feet, and macrodactyly of the left foot. Clinical suspicion of Klippel-Trénaunay-Weber syndrome was set due to the presence of lymphangiomas. In 2019, NGS gene panel testing TSOE was performed on blood, but no pathogenic variants in the *PIK3CA* gene were detected. In 2023, the TSO500 gene panel was performed on DNA extracted from skin biopsy and lymphangioma tissue samples obtained from the affected area from the left hip. Analysis of the affected skin biopsy revealed a heterozygous mosaic pathogenic variant in the *PIK3CA* gene, specifically the p.Glu542Lys pathogenic variant, with VAF of 0.8% at a sequencing depth of 1236x. Similarly, analysis of DNA extracted from the affected lymphangioma tissues identified the same *PIK3CA* p.Glu542Lys pathogenic variant, with a VAF of 7% at a sequencing depth of 1377x in one sample, and a VAF of 9% at a sequencing depth of 1,144x in the other sample.

## 4 Discussion

PRD are rare affecting approximately 14 births per 1 million (Nadjkovic and Lonabaugh, 2023). These conditions are known to exhibit somatic mosaicism, where pathogenic *PIK3CA* variants manifest solely in a subset of cells within the affected tissues. Conventional methods like Sanger sequencing and first generation sequencing methods cover the entire *PIK3CA* coding region but lack the depth to detect low-frequency mosaic variants in PRD, especially when tissue samples are limited (Keppler-Noreuil et al., 2015). Acquiring suitable tissue samples for genetic analysis becomes problematic, particularly in cases where accessibility to affected tissues is limited or collecting the biopsy from the patient poses risks to the patient (Mirzaa et al., 1993; Mirzaa et al., 2016). Thus, the adoption of comprehensive diagnostic approaches, notably NGS methods, stands as an essential alternative to conventional methods, addressing the limitations associated with PRD (Chang et al., 2017). These advanced techniques offer enhanced sensitivity and accuracy, facilitating the identification of low-level mosaic variants and providing a thorough assessment of the spectrum of pathogenic variants linked to PRD, while also requiring a specific NGS methods and bioinformatics pipeline for variant calling (Mirzaa et al., 2016; Gazzin et al., 2023). Considering the variability of mosaicism, our experience suggests that using comprehensive NGS methods such as the TSO500 NGS gene panel is recommended, as it provides better coverage for the targeted *PIK3CA* region. Clinical diagnosis of PRD proves challenging due to the disease variability, overlapping phenotypes, tissue mosaicism, and the limitations of diagnostic methods, all of which contribute to difficulties in confirming pathogenic *PIK3CA* gene variants (de Kock et al., 2023).

In the literature, several “hotspot” gene variants in the *PIK3CA* gene are frequently altered, e.g., p.Glu542Lys, p.Glu545Lys, p.His1047Arg, p.His1047Lys and some “non-hotspot” gene variants such as p.Gly726Lys and p.Gly914Arg (Mirzaa et al., 1993; 2016). Our study identified one “hotspot” pathogenic *PIK3CA* gene variant (p.Glu542Lys) and two pathogenic/likely pathogenic gene variants (p.Gly914Arg and p.Gln546Lys) in the *PIK3CA* gene classified as “non-hotspot.” The residues p.Glu542Lys and p.Gln546Lys, located within the helical domain, are gain-of-function variants that enhance the catalytic activity of the p110α subunit. These variants likely interfere with the p110α-p85α interaction, altering regulatory control. In contrast, the residue p.Gly914Arg is situated in the kinase domain of the *PIK3CA* gene (Gabelli et al., 2010; Jenkins et al., 2023). The first detected *PIK3CA* variant, p.Gly914Arg, has been previously reported mainly in patients with megalencephaly-capillary malformation-polymicrogyria syndrome (Chang et al., 2017). Additionally, this variant has also been documented in diverse clinical presentations, including a seven-year-old male with congenital macrocephaly and right-lateralized overgrowth (Maguolo et al., 2018) and a 28-year-old female with segmental overgrowth who experienced headaches and had unilateral vestibular schwannoma and meningiomas (Mills et al., 2018). The second pathogenic *PIK3CA* gene variant, p.Gln546Lys, has been reported in a newborn who had lymphatic malformation of the tongue (Zenner et al., 2020). The third *PIK3CA* variant, p.Glu542Lys, has been reported in an 8-month-old male patient exhibiting bilateral hydrocele, lipomatous infiltration in muscles and internal structures, vascular malformations, kidney abnormalities, and significant delays in gross motor development. Another case involves an 11-month-old male patient with fibro-adipose overgrowth, overgrowth of the foot, macrodactyly of the left T1-3, and a 43-year-old female patient presenting with an ovarian cyst, overgrowth of the right foot, thigh, and hand, tongue masses, lipomatous infiltration in the liver, spleen, mediastinal and left foot T1-4 paraspinal areas, vascular malformation in the proper abdominal back and groin, as well as multiple haemangiomas, kidney cysts, and cutaneous blebs (Keppler-Noreuil et al., 2014).

Macrodactyly, a rare congenital condition, is less frequently observed in the foot than in the hand. Typically, the second toe is the more commonly affected digit in cases of foot macrodactyly (Shen et al., 2022). Patient 2 in our cohort exhibited isolated macrodactyly in the right foot, explicitly affecting T2-3. Krishnamurthy et al. reported a PRD patient involving the index finger and thumb with isolated macrodactyly in the right hand. This patient underwent surgical intervention and received alpelisib treatment to target areas where surgery alone could not sufficiently address the issue (Krishnamurthy et al., 2023).

*PIK3CA* pathogenic gene variants are also associated with various types of cancers such as endometrial, breast, ovarian, bladder, colorectal Wilms tumour, hepatoblastoma, adrenocortical carcinoma and others (Arafeh and Samuels, 2019). However, Faivre et al. found that cancers observed in PRD patients may not always be directly linked to the syndrome itself, and further research is essential to understand better the relationship between PRD and an increased risk of cancer (Faivre et al., 2023).

PRD treatment options are limited and focus on managing symptoms and complications. Management involves a multidisciplinary team of specialists, including geneticists, paediatricians, dermatologists, orthopaedic surgeons, and neurologists. Orthopaedic interventions, dermatological interventions, physical and occupational therapies, and targeted therapies are being investigated as potential treatment options (Mirzaa et al., 1993). Clinical studies have shown promising results in reducing overgrowth and improving symptoms, but further research is needed to establish the long-term safety and efficacy of these targeted therapies (Douzgou et al., 2022). Alpelisib received approval from the US Food and Drug Administration (FDA) on 5 April 2022, for use in patients aged 2 years and older (Singh et al., 2023). Before alpelisib, there were no FDA-approved drug treatment options for PRD. In their overview, Nadjkovic and Lonabaugh detailed alpelisib clinical applications, efficacy and potential side effects, including hypersensitivity, cutaneous adverse reactions, hyperglycaemia, pneumonitis, diarrhoea, and embryo-fetal toxicity (Nadjkovic and Lonabaugh, 2023). In Estonia, various mTOR inhibitors, such as sirolimus/rapamycin, have been used to slow the progression of existing lesions and overgrowths. Customised treatment plans tailored to each patient’s needs are necessary to optimise patient outcomes.

In conclusion, detecting phenotypically related pathogenic variants associated with somatic mosaicism poses a challenge when relying solely on conventional diagnostic approaches. NGS methods provide more comprehensive genomic profiling, precisely determining variant allele frequency and facilitating the identification of somatic changes. The three cases presented in this study emphasise the significance of employing comprehensive genomic profiling methods, such as NGS-based techniques, to analyse all relevant pathogenic gene variants associated with phenotypic overlaps in affected tissue(s). This approach can enhance the accuracy and diagnostic yield in patients with similar presentations, ultimately improving patient care and management.

## Data Availability

Datasets from Tartu University Hospital are not publicly available. Genetic sequencing data presented in the publication originates from clinical testing. Requests to access them should be directed to Mikk Tooming, email: mikk.tooming@kliinikum.ee.
