# Supplementary material for: Uncovering somatic mosaic variants of PIK3CA-related overgrowth disorders – three cases with different clinical presentations
Source: Front Genet. 2025 Jan 13;15:1484651. doi: 10.3389/fgene.2024.1484651 (PMC11769973; doi:10.3389/fgene.2024.1484651)
Supplement: Supplementary file 1 [file DataSheet1.pdf]

CARE Checklist

1. Title – The diagnosis or intervention of primary focus followed by the words “case report”.

Uncovering somatic mosaic variants in PIK3CA-related overgrowth disorders: Case report of three patients with distinct clinical presentations

2. Key Words
– 2 to 5 key words that identify diagnoses or interventions in this case report (including "case report").

PIK3CA-related overgrowth syndrome, PIK3CA somatic mosaic variants,
overgrowth syndrome, next-generation sequencing-based genomic profiling, case report

3. Abstract – (structured or unstructured)
Introduction – What is unique about this case and what does it add to the scientific literature?
The patient’s main concerns and important clinical findings.
The primary diagnoses, interventions, and outcomes.
Conclusion – What are one or more “take-away” lessons from this case report?

All patients in this study were diagnosed with *PIK3CA*-related disorders (PRDs, OMIM *171834). The cases presented are particularly noteworthy due to their diverse clinical manifestations, the broad spectrum of *PIK3CA* somatic pathogenic variants detected, and the application of advanced genomic profiling techniques that are not typically used in routine PRD diagnostics. This study also documents a rare instance of PRD in Estonia, a condition underreported in scientific literature.

This research enhances our understanding of the clinical variability of PRDs, highlights the diagnostic challenges posed by somatic mosaicism, and underscores the importance of comprehensive genomic tools in accurately identifying pathogenic variants. The study illustrates significant variability in clinical symptoms, ranging from mild intellectual disability, physical overgrowth, macrocephaly, and macrodactyly of the foot to complex vascular and lymphatic malformations. Identifying specific pathogenic variants in the *PIK3CA* gene, including both “hotspot” and “non-hotspot” variants, has contributed to a better understanding and managing these conditions.

The importance of this study lies in its demonstration of the value of comprehensive genomic profiling in cases where conventional diagnostic methods fail due to somatic mosaicism. It also emphasizes the need for personalized approaches in managing the diverse clinical manifestations of PRDs, given the variability in severity even among patients with identical genetic alterations.

4. Introduction
– Briefly summarizes why this case is unique and may include medical literature references.

The cases are unique due to the application of advanced genomic profiling in diagnosing PRDs, a rare group of disorders characterized by somatic mosaicism and challenging clinical

presentations. The study emphasizes the limitations of conventional diagnostic methods and advocates for using comprehensive genomic tools for accurate diagnosis and better patient management.

5. Patient Information
De-identified patient specific information.
Primary concerns and symptoms of the patient.
Medical, family, and psychosocial history including relevant genetic information.
Relevant past interventions and their outcomes.

De-identified patient-specific information is provided, focusing on genetic analysis and clinical presentations. Previous diagnostic efforts included genetic testing, but challenges in confirming the diagnosis due to the mosaic nature of the disease were noted.

6.Clinical Findings
– Describe significant physical examination (PE) and important clinical findings.

Patient 1: notable findings include macrocephaly, cerebral and cerebellar hemisphere asymmetry, and thickened corpus callosum.
Patient 2: Persistent syndactyly and advanced bone age in the affected toes, with typical cerebral structures.
Patient 3: Extensive lymphangiomas, syndactyly and abnormal foot growth

7. Timeline – Historical and current information from this episode of care organized as a timeline (figure or table).

All information is described in table 1 and figures 1, 2 and 3.

Patient 1: Symptoms onset > 2 months (initial imaging) > 7 years (continued imaging) > genetic diagnosis at fibroblast culture.
Patient 2: 1.5 years (initial foot X-ray) > 2.5 years (follow-up imaging) > 4 years (brain MRI and genetic diagnosis)
Patient 3: Antenatal (cystic formation identified) > 4.5 years (lymphangioma diagnosis) > genetic testing and confirmation of diagnosis)

8. Diagnostic Assessment
Diagnostic methods (PE, laboratory testing, imaging, surveys).
Diagnostic challenges.
Diagnosis (including other diagnoses considered).
Prognostic characteristics when applicable.

Diagnostic methods include physical examination, imaging (MRI, X-ray), and advanced genomic testing through next-generation sequencing. The diagnostic challenges in the mosaic nature of PRDs make it difficult to detect pathogenic variants through standard diagnostic methods, necessitating more sensitive and comprehensive approaches. PRD was confirmed in all three cases, with different *PIK3CA* pathogenic gene variants identified. PRD prognosis depends on the severity and location of the overgrowth and malformations, with some patients requiring ongoing management for complications.

9. Therapeutic Intervention

Types of therapeutic intervention (pharmacologic, surgical, preventive).
Administration of therapeutic intervention (dosage, strength, duration).
Changes in therapeutic interventions with explanations.

The therapeutic interventions were not focused of this study.

10. Follow-up and Outcomes
Clinician- and patient-assessed outcomes if available.
Important follow-up diagnostic and other test results.
Intervention adherence and tolerability. (How was this assessed?)
Adverse and unanticipated events.

In this case series, the clinician-assessed outcomes primarily focused on identifying the *PIK3CA* variants and the phenotypic manifestations in each patient. This included documentation of clinical symptoms such as macrocephaly, macrodactyly, and lymphangiomas and their progression over time. Patient outcomes varied, with some experiencing more severe symptoms like intellectual disability and structural brain abnormalities, while others exhibited more isolated physical symptoms like macrodactyly.
For all three patients, follow-up diagnostic tests included imaging studies and/or genetic testing. In Patient 1, MRI scans at various ages showed persistent and worsening neurological abnormalities, such as megalencephaly and asymmetric brain development. In Patient 2, X-ray and MRI studies demonstrated the ongoing disproportionate growth of the affected foot, consistent with PRD. Patient 3 follow-up involved genetic testing of different tissue samples, which confirmed the presence of the *PIK3CA* p.Glu542Lys variant in both skin biopsy and lymphangioma tissue, underscoring the importance of testing affected tissues for accurate diagnosis. The repeated imaging and biopsy procedures required for precise diagnosis were critical in monitoring the disease progression and ensuring the correct diagnosis.

11. Discussion
Strengths and limitations in your approach to this case.
Discussion of the relevant medical literature.
The rationale for your conclusions.
The primary "take-away" lessons from this case report (without references) in a one paragraph conclusion.

The strengths of this case series include the comprehensive genetic and clinical assessment of PRD patients, which allowed for the identification of rare *PIK3CA* variants and their associated phenotypic presentations. Advanced genomic techniques, such as next-generation sequencing and the testing of affected tissues, were pivotal in overcoming the challenges of detecting low-level mosaic variants. This approach also provided valuable insights into the variability of PRD presentations within a small cohort, enhancing understanding of the disease spectrum.
Limitations of this study include the small sample size, which limits the generalizability of the findings. Additionally, the case series primarily focuses on the diagnostic aspects of PRDs, with less emphasis on long-term clinical outcomes, treatment efficacy, or quality-of-life impacts for the patients.
The findings from this case series align with existing literature on *PIK3CA*-related overgrowth disorders (PRDs), shedding light on the challenges of diagnosing these conditions due to the mosaic nature of the *PIK3CA* variants. It is crucial to identify both "hotspot" and "non-hotspot" variants in the *PIK3CA* gene, as standard diagnostic methods may fail to target "non-hotspot" genetic variants, making their detection impossible. The detected variants are consistent with

previous studies, which have documented a range of phenotypic manifestations. For instance, the p.Glu542Lys variant is frequently associated with more severe overgrowth syndromes, as supported by literature from Keppler-Noreuil et al. (2015). This study underscores the importance of comprehensive genomic profiling in diagnosing PRDs, as traditional methods may overlook low-level mosaic variants. This is exemplified by successfully identifying *PIK3CA* variants in affected tissues, which would likely have been missed using standard, less comprehensive blood-based approaches. Embracing this approach enhances diagnostic accuracy and informs better management and care strategies for patients with PRDs.

12. Patient Perspective – The patient should share their perspective on the treatment(s) they received.

Based on the etiological information we have provided and the confirmed clinical diagnosis of PRDs in our patients, various mTOR inhibitors could be used to slow the progression of the existing lesions and overgrowths. Treatment has not yet been started for our patients, which is why we were unable to provide the patients' perspectives on the treatment.

13. Informed Consent – The patient should give informed consent. (If not, explain).

Written informed consent was obtained from the two patients to describe the clinical symptoms and radiological findings in detail. The study was approved by the Research Ethics Committee of the University of Tartu (278/T-19, 288/M-17, 340/M-17, 372/M-8 and 387/M-15). It was conducted following the rules of the Declaration of Helsinki (World Medical Association Declaration of Helsinki: Recommendations Guiding Physicians in Biomedical Research Involving Human Subjects, 1997).
